# Compositional Development of *Bifidobacterium* and *Lactobacillus* Microbiota Is Linked with Crying and Fussing in Early Infancy

**DOI:** 10.1371/journal.pone.0032495

**Published:** 2012-03-05

**Authors:** Anna Pärtty, Marko Kalliomäki, Akihito Endo, Seppo Salminen, Erika Isolauri

**Affiliations:** 1 Department of Pediatrics, Turku University Hospital, University of Turku, Turku, Finland; 2 Department of Biochemistry and Food Chemistry, Functional Foods Forum, University of Turku, Turku, Finland; University of Hyderabad, India

## Abstract

**Objectives:**

Our aim was to establish whether there is an interconnection between the compositional development of the gut microbiota and the amount of fussing and crying in early infancy.

**Methods:**

Behavioral patterns of 89 infants during the 7^th^ and 12^th^ week of life were recorded in parental diaries. Total distress was defined as the sum of daily amounts of crying and fussing. Infants' gut microbiota profiles were investigated by several molecular assays during the first six months of life.

**Results:**

The median (range) duration of total distress among the infants was 106 (0–478) minutes a day during the 7^th^ and 58 (0–448) minutes a day during the 12^th^ week. The proportion of *Bifidobacterium* counts to total bacterial counts was inversely associated with the amount of crying and fussing during the first 3 months of life (p = 0.03), although the number of *Bifidobacterium breve* was positively associated with total distress (p = 0.02). The frequency of *Lactobacillus* spp. at the age of 3 weeks was inversely associated with total infant distress during the 7^th^ week of life (p = 0.02).

**Conclusions:**

*Bifidobacterium* and *Lactobacillus* appear to protect against crying and fussing. Identification of specific strains with optimal protective properties would benefit at-risk infants.

## Introduction

Infants communicate and express discomfort by crying. One infant in four manifests with abundant cry-fuss or colic cry warranting medical evaluation [1]. The most widely cited criterion is Wessel's rule of three, which characterizes infantile colic as paroxysmal, excessive and inconsolable crying, its duration exceeding 3 hours a day for 3 days or more in any one week [2]. It is not clear, however, whether infants with colic constitute a clinical entity or an arbitrary upper limit of normal crying [3].

Moreover, despite more than 50 years of research into the origins of infant crying, the precise cause of excessive crying remains unresolved. Regardless of its cause, infant crying is a source of frustration to parents and is associated with maternal emotional distress and loss of the sense of parental competence [4], [5].

Excessive crying in an otherwise healthy infant coincides with several maturational processes which take place particularly in the gastrointestinal tract in response to massive antigen challenges by microbial colonization and food intake. Indeed, the principal attempts to control excessive crying have focused on various dietary regimens and modulation of the gut microbiota [6]–[8]. Initial colonizers of the newborn gastrointestinal tract are facultative anaerobic bacteria, followed by strict anaerobic populations of *Bifidobacterium*, *Clostridium* and *Bacteroides* [9]. Deviating colonization processes have been proposed to underlie the development of infant colic, while their impact on infant fussing or crying remains elusive [10]–[13].

The objective of the study was to characterize the interconnection between gut microbiota and crying in early infancy. We focused on the entire spectrum of crying as opposed to the most often assessed colic cry, as we need to appreciate the full scope of distress behaviour causing concern among a significant proportion of parents [1]. For this purpose, we recorded behavioral patterns of infants and analyzed their gut microbiota on two fronts, firstly by quantitative polymerase chain reaction (qPCR) and fluorescent in situ hybridization (FISH) assays and secondly by PCR-denaturing gradient gel electrophoresis (PCR-DGGE) during the first six months of life.

## Materials and Methods

### Ethics Statement

The study was approved by the committees on ethical practice in Turku University Hospital and the Health Office of Turku. Written informed consent was obtained from the children's parents.

### Subjects and samples

**Figure 1:**
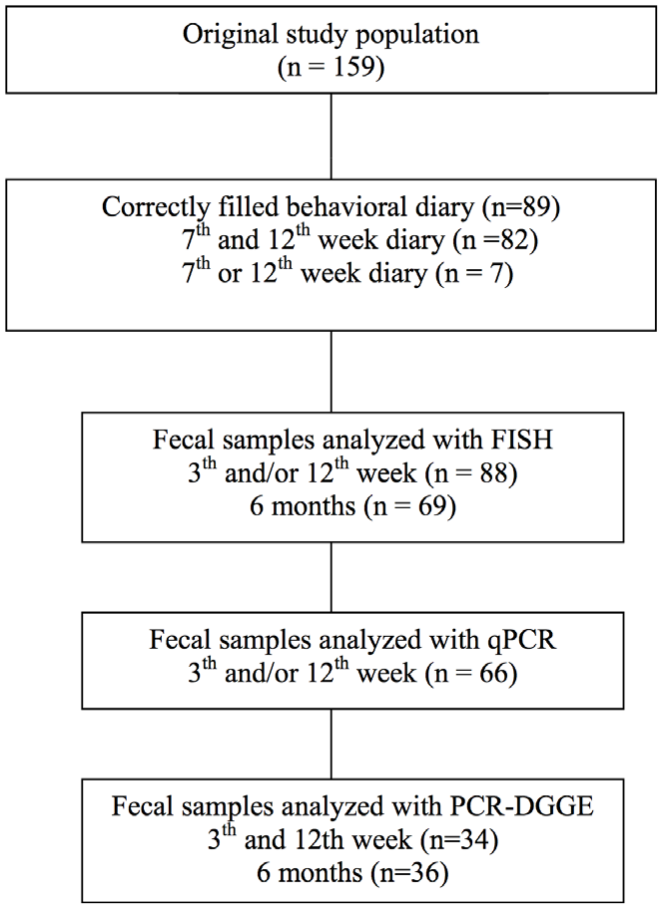
Flow chart of the procedure.

The subjects included in this study are participants in an ongoing randomized, double-blind, placebo-controlled study involving perinatal *Lactobacillus rhamnosus* GG (ATCC 53103) intervention (http://www.clinicaltrials.gov/ct/gui/show/NCT167700) as described in detail elsewhere [14], [15]. The focus here however, was not the probiotic intervention but the early microbiota composition. In brief, the original study population comprised 159 infants who had at least 1 close relative with atopic dermatitis, allergic rhinitis or asthma. The mothers of these children were recruited in the antenatal clinics of the city of Turku (population 170,000) and randomized in double-blind, placebo-controlled manner to receive 1×10^10^colony-forming units of *Lactobacillus rhamnosus* GG (ATCC 53103) or placebo (microcrystalline cellulose) capsules once a day for 4 weeks before expected delivery. After delivery, the capsule contents were given either to the children, mixed with water, or continuously to the mother, if breast-feeding, for 6 months. Altogether 89 infants fulfilled the inclusion criteria for the present study: a correctly filled parental diary of the infant's behavior and status during the 7^th^ and/or 12^th^ week of life (in 82 cases on both occasions) (Figure 1).

The parents recorded their infants' behavior patterns (sleeping time, time awake and content, fussing, colic-type cry, other cry, and feeding), vomits, stools with their consistency, and skin condition, using a modified 24-hour Barr chart [16] on seven consecutive days during the 7^th^ and 12^th^ week of life. The smallest recording unit on the chart was 15 minutes. Fussing was defined as a state of irritability, “not quite crying but not awake and content” [16]. Other cry was defined as crying responsive to intervention (feeding, diaper change, carrying, sucking a pacifier), and colic-type cry as a cry not responsive to such intervention. Total distress, the amount of crying and fussing, was the sum of these various modes as reported by the parents. The study population was not classified on the basis of the crying amount. Instead crying symptoms was taken as a continuous variable among the entire study population. The children were clinically examined at three-month intervals, always by the same physician (MK). A fecal sample was taken at 3 weeks, 3 months and 6 months, either by nursing staff at a scheduled visit or by the parents immediately before it. In the latter case, the sample was stored at 4°C and delivered to the hospital within 24 hours.

### DNA Isolation and qPCR assay

Faecal samples were stored at −80°C until analyzed. Samples were pre-treated and DNA was extracted using an automated KingFisher DNA extraction system (Thermo Fisher Scientific Oy, Vantaa, Finland) and InviMag Stool DNA kit (Stratec Molecular, Berlin, Germany), as previously described [17]. The standard DNA for quantitative PCR (qPCR) was prepared as described previously [17]. All DNA Samples were stored at −20°C until analyzed. Quantitative PCRs were conducted as previously described [18], [19]. PCR amplification and detection were performed with an ABI PRISM 7300-PCR sequence detection system (Applied Biosystems, Foster City, CA).

### FISH assay

Bacterial cells were harvested and fixed and FISH performed with fluorophore (indocarbocyanine Cy3) labeled oligonucleotide probes, as described previously [20]. Total cell numbers were determined by staining a nucleic acid stain 4′,6-diamidino-2-phenylindole (DAPI). Cells were counted visually using a Olympus SZX9 epifluorescence microscope (Olympus Optical Co LTD, Japan).

### PCR-DGGE

The composition of *Lactobacillus* spp. and *Bifidobacterium* spp. in the fecal samples was analyzed by PCR-DGGE. PCR amplification of DNA of each bacterial group was done as described previously [21]. Amplification was confirmed by gel electrophoresis in 1.0% agarose. DGGE analysis of each PCR product was conducted with a DCode System (Bio-Rad Laboratories, Hercules, CA, USA), as previously described [22]. DNA bands were re-amplified using the same primer set as for generating the DGGE samples. The PCR products were purified and sequenced according to the method previously described [22]. Sequences were compared with known sequences at GenBank by BLAST analyses. The detection limit was approximately 10^5^ CFU/g of feces. The nucleotide access numbers for the sequences determined are AB671744-AB671754.

### Statistics

The clinical characteristics of the study subjects for continuous variables are given as mean values (standard deviations (SDs)) or medians (range) for normal and non-normal distribution, respectively, and as numbers and proportions for categorical variables. The variables amount of total distress, proportion of *Bifidobacterium* counts to total bacterial counts (%) and proportion of *Lactobacillus* counts to total bacterial counts (%) were square-root transformed in the statistical analyses.

Associations between continuous outcome variables, amount of crying and fussing and gut microbiota, were studied by mixed model repeated measures analysis (samples at 3 weeks and 3 months of age). Univariate associations between the amount of crying and fussing during the first 3 months of life and numbers of gut microbiota bacteria at 6 months were studied using linear regression analysis.

Unvariate associations between amount of crying and dichotomous variables were studied using independent samples t-test. The association between the intervention and dichotomous *Lactobacillus* PCR-DDGE measurement was studied using a generalized linear mixed model with binary response variable. Subject was used as random effect and intervention, time and interaction between intervention and time as independent variables. As the effect of intervention differed at the three time points the results for each time point are presented separately. A *P* value<0.05 was considered statistically significant.

## Results

The clinical characteristics of the study subjects are presented in Table 1. The median (range) duration of total infant distress was 106 (0–478) minutes a day during the seventh week and 58 (0–448) minutes a day during the twelfth week. The median (range) duration of colic-type crying was 6 (0–118) minutes per day during the seventh week and 2 (0–69) during the twelfth week. Other infant crying characteristics are presented in Table 2.

**Table 1:** Clinical characteristics of the study subjects.

	n = 89
Vaginal delivery	73 (82)
Male/female	59/30
Gestational age at birth (wk)	40 (35–42)
Birth weight (g)	3601 (480)
Birth length (cm)	51 (45–55)
Apgar score at 5 min	9 (8–10)
Exclusively breast-fed (months)	2.5 (0.1–6.0)
Total duration of breastfeeding (months)	5.5 (0.5–16)
Antibiotic treatment during the first 6 months of life*	25 (29)
*Lactobacillus rhamnosus* GG supplementation during the first 6 months of life	42 (47)

Results are given as mean (SD) or median (range) or as number (%) of subjects, if not otherwise stated.

*n = 85.

**Table 2:** The amount (minutes/day; median with range) of fussing and crying, and total distress reported by parents during the 7^th^ and 12^th^ weeks of life.

	7^th^ week	12^th^ week
Fussing*	65 (0–255)	31 (0–319)
Other cry†	30 (0–124)	15 (0–154)
Colic-type cry‡	6 (0–118)	2 (0–69)
Total distress	106 (0–478)	58 (0–448)

*Fussing was defined as a state of irritability, “not quite crying but not awake and content”.

† Other cry was defined as crying responsive to intervention (feeding, change of diaper, carrying, sucking a pacifier); colic-type cry was defined as a cry not responsive to such intervention.

‡ The sum of colic type, other cry, and fussing reported by parents.

During the first 6 months of life 47% of the study population received *Lactobacillus rhamnosus* GG supplementation. The use of the probiotic had no effect on infant total distress during the 7^th^ or 12^th^ week of life (p = 0.74 and p = 0.82 respectively). There was no statistically significant difference in total distress between children with or without antibiotic treatment during the 7^th^ or 12^th^ week of life (p = 0.13 and p = 0.62, respectively). At the age of 7 and 12 weeks 90% and 78% of the infants were breast-fed. The total distress was not statistically different between breast-fed and formula-fed infants at the ages of 7 and 12 weeks (p = 0.08 and p = 0.45, respectively).

### FISH and qPCR

At the age of 3 months the mean (95% CI) total count of *Bifidobacterium*, as measured by FISH, was 9.4 (9.2–9.6) log cells/g and the mean (95% CI) proportion of *Bifidobacterium* counts to total bacterial counts was 61% (50–72). This proportion was inversely associated with the amount of crying and fussing during the first 3 months of life (p = 0.03). In contrast, *Bifidobacterium breve* behaved contrary to this general *Bifidobacterium* pattern: the amount of *Bifidobacterium breve*, as analyzed by qPCR, was found to be associated with the amount of total distress (p = 0.02). Moreover, the proportion of different bacterial groups counted by FISH (i.e. the sum of the counts of *Bacteroides-Prevotella* group, *Clostridium histolyticum* group, *Bifidobacterium* genus and *Lactobacillus-Lactococcus-Enterococcus* group) to total bacterial counts tended to be inversely associated with the amount of crying and fussing (p = 0.06).

At the age of 6 months, after the peak period of crying, crying and fussing documented at 3 months still paralleled the gut microbiota composition, the total number of *Bifidobacterium* (p = 0.03) and *Lactobacillus* (p = 0.008) being inversely associated with the total distress experienced during the first 3 months. Again, the proportion of the total counts of different bacterial groups to total bacterial counts was inversely associated with the amount of crying and fussing (p = 0.04).

### PCR-DGGE

PCR-DGGE was performed to further assess the composition of *Bifidobacterium* with the samples available. *Bifidobacterium* spp. was detected in almost all samples tested, the frequencies being 82%, 85% and 97% of the samples analyzed at the age of 3 weeks, 3 months and 6 months, respectively. Either *B. longum* (accession no. AB671752) or *B. breve* (AB671749) was predominantly seen at the age of 3 weeks, and *B. longum*, *B. bifidum* (AB671750), *B. catenulatum/pseudocatenulatum* (AB671751) and *B. breve* at the age of 6 months (Table 3). The frequency of B. *longum* at the age of 3 weeks was inversely associated with total distress during the 7^th^ week of life, although the association did not reach statistical significance (p = 0.20).

**Table 3:** The frequency of *Bifidobacterium spp*. by PCR-DGGE during the first 6 months of life.

	3^th^ week	12^th^ week	6^th^ month
*B. breve*	29% (10/34)	35% (12/34)	25% (9/36)
*B. adolescentis*	9% (3/34)	6% (2/34)	14% (5/36)
*B. bifidum*	12% (4/34)	24% (8/34)	42% (15/36)
*B. angulatum*	N.D.	3% (1/34)	N.D.
*B. longum*	44% (15/34)	59% (20/34)	72% (26/36)
*B. catenulatum* group*	18% (6/34)	18% (6/34)	31% (11/36)

* *B. catenulatum* group includes *B. catenulatum* and *B. pseudocatenulatum*.

N.D. not detected.

The *Lactobacillus* spp. was detected by PCR-DGGE in 24, 56 and 75% of the infants at 3 weeks, 3 months and 6 months, respectively. The prevalence of *Lactobacillus* spp. at the age of 3 weeks was inversely associated with total infant distress during the 7^th^ week of life (p = 0.02). The prevalence of *Lactobacillus* species differed significantly between the probiotic and placebo groups at the age of 3 months, (80% and 37%, respectively, p = 0.01), and at the age of 6 months, (95% and 53%, respectively, p = 0.02), but not at 3 weeks (22% and 25%, respectively, p = 0.85). DGGE profiles indicated that the *Lactobacillus rhamnosus* group (accession no. AB671744) was the predominant species in almost all samples tested, while other species (accession no. AB671745-AB671648) were detected only occasionally.

## Discussion

The data obtained from the present study demonstrate for the first time that low proportions of *Bifidobacterium*, regarded as key biological markers of the healthy breast-fed infant's gut microbiota, as well as a low prevalence of *Lactobacillus* appear to be risk factors for infant crying and fussing. The results further suggest that specific *Bifidobacterium* species exert distinct effects on the infant's total distress, *Bifidobacterium longum* being potentially protective, *Bifidobacterium breve*, again, carrying a heightened risk of distress. Finally, the alterations in the gut microbiota persisted although the amount of crying decreased. Such observations indicate a need to evaluate of long-term sequels of deviant gut microbiota composition associated with infant distress.

Our study differed in certain respects from earlier investigations. Firstly, these focused on infants crying in extreme amounts while the present study on the entire spectrum of crying. In colic infants, altered intestinal fatty acid profiles, decreased numbers of lactobacilli and increased counts of anaerobic gram-negative and coliform bacteria as well as increased fecal calprotectin levels have been reported [11]–[13], [23]. Secondly, in contrast to earlier studies [10]–[12], which have used culture methods to identify bacteria in stool samples, we used more precise quantitative PCR, FISH and PCR-DGGE assays. Moreover it is of note that our result, obtained by culture-independent methods, was not limited to one method, since both quantitative PCR and PCR-DGGE assays pointed to *Bifidobacterium breve* as a risk factor.

Our original findings here link total distress to a decreased proportion of *Bifidobacterium* and a lower frequency of *Lactobacillus*, i.e. to changes in the gut microbiota. Once again, it should be pointed out that the crying amount among the infants in our cohort represented the whole spectrum of crying, not the extreme end of it, colic, as in previous works. It is thus interesting to speculate that changes in the gut microbiota might have a pivotal effect on the amount of crying, regardless of whether the child fulfills the criteria of colic or not. In that case, colic crying would rather constitute an arbitrary upper limit of normal crying than a separate clinical entity. Such a conception is also supported by earlier clinical observations. Indeed, there are several similarities between the crying behavior of infants with and without colic [24]. As an example, healthy children at 6 weeks of age cry on average for 2 hours and 45 minutes a day, which is very close to the lowest limit of daily crying in a colic child [25].

Our results confirm the establishment of the gut microbiota as a gradual process; *Bifidobacterium* appear after birth and within a few weeks emerge as the dominant bacterial group, while the number of *Lactobacillus* increases during the entire follow-up period. Our analysis of *Lactobacillus* spp. indicated that the *Lactobacillus rhamnosus* group was the predominant species in the population, irrespective of the intervention. This finding might explain why the probiotic intervention had no effect on crying amount. Unfortunately, our molecular assays did not allow characterization of *Lactobacillus rhamnosus* at strain level. We can only assume that the detected DNA might have originated, besides from the study product in the probiotic group, also from dairy products containing different strains of *Lactobacillus rhamnosus*.

There are data on record to demonstrate that individual specific *Bifidobacterium* species and even different strains exert distinct effects on immunity and disease risk [26], [27]. To take an example, allergic infants are most often colonized by *Bifidobacterium adolescentis*, while healthy infants harbor mainly *Bifidobacterium bifidum* strains [28]. Our findings here suggest that early colonization by *Bifidobacterium longum*, the species most widely found in human breast milk [29], may have a positive impact on infant distress. On the other hand, the amount of *Bifidobacterium breve* was found to be associated with an increasing amount of daily crying and fussing during the first months of life. A recent experimental study in gnotobiotic mice gives some indication of the effects of these two different *Bifidobacterium* species on the developing immune system [30]. *Bifidobacterium longum* strains isolated from infants' fecal microbiota were able to induce strain-specific effects on both systemic and intestinal immunity while *Bifidobacterium breve* strains were found to be only weak or nil inducers [30]. Despite these profound differences between these two *Bifidobacterium* strains for the early development of the immune system, the precise mechanisms by which these strains may impact on infant's crying behavior need to be clarified.

Our findings link the composition of the gut microbiota to fussing and crying during early infancy in demonstrating strain-specific effects of gut microbiota on infant distress. Future detailed elucidation of these effects may facilitate the development of novel preventive and therapeutic options against this common and often nerve-racking problem.
